# Development of an age-specific paediatric 2-[^18^F]fluoro-2-deoxy-D-glucose PET template aligned with paediatric brain development regularities: enhancing epileptogenic zone localization in drug-resistant epilepsy

**DOI:** 10.1093/braincomms/fcag280

**Published:** 2026-07-16

**Authors:** Huan Ma, Xin Wen, Haizhou Qiao, Xiaojuan Tian, Keyu Zhang, Guanyun Wang, Ying Kan

**Affiliations:** Nuclear Medicine Department, Beijing Friendship Hospital, Capital Medical University, Beijing 100050, China; Nuclear Medicine Department, Beijing Friendship Hospital, Capital Medical University, Beijing 100050, China; Department of Nuclear Medicine, Beijing RIMAG Medical Imaging Center, Beijing 100020, China; Department of Neurology, Beijing Children’s Hospital, Capital Medical University, Beijing 100045, China; Nuclear Medicine Department, Beijing Friendship Hospital, Capital Medical University, Beijing 100050, China; Nuclear Medicine Department, Beijing Friendship Hospital, Capital Medical University, Beijing 100050, China; Nuclear Medicine Department, Beijing Friendship Hospital, Capital Medical University, Beijing 100050, China

**Keywords:** epilepsy, positron emission tomography (PET), paediatric-specific SPM template

## Abstract

This study aims to systematically analyse developmental regularities of brain glucose metabolism in children covering the whole paediatric age range and establish age-specific paediatric 2-[^18^F]fluoro-2-deoxy-D-glucose PET brain templates, thereby improving the accuracy of neuroimaging lesion localization in paediatric populations. A retrospective study was conducted, including a pseudo-control group and an epilepsy group. The pseudo-control group data were used to analyse the cerebral glucose metabolism and establish age-specific paediatric statistical parametric mapping templates. Semi-quantitative parameters (mean standardized uptake value, maximum standardized uptake value) were calculated for each region, and standardized uptake ratio was compared across different reference regions. Age-related trends of these metabolic parameters were analysed to derive paediatric cerebral metabolic developmental patterns. 2-[^18^F]fluoro-2-deoxy-D-glucose PET images of patients with epilepsy were performed using both the established paediatric template and the standard adult template. Taking surgical resection and electrocorticography results as the gold standard, the accuracy of epileptogenic zone localization by the two templates was compared. The mean standardized uptake value and maximum standardized uptake value in the pseudo-control group showed a significant positive correlation with age, with no significant differences between sex or hemispheres (*P* > 0.05). In the internal and external validation cohorts, the diagnostic accuracy of the paediatric statistical parametric mapping template for epileptogenic zone localization was 79.0%, which was significantly higher than 72.5% of the adult template (*P* = 0.033). Stratified analysis showed that the paediatric template had consistent advantages across sex and superior performance in epileptogenic zone localization of frontal and temporal lobes. The advantage of the paediatric template was consistent in both internal and external validation cohorts. The age-specific paediatric statistical parametric mapping template has superior overall diagnostic efficacy in epileptogenic zone localization of paediatric drug-resistant epilepsy, with stable advantages across sex and unique value in specific brain regions. It can overcome the limitations of adult templates in adapting to paediatric brain development characteristics, providing a more accurate and reliable tool for preoperative epileptogenic zone localization in paediatric drug-resistant epilepsy.

## Teaser

Ma Huan *et al*. built age-specific paediatric [^18^F]FDG PET templates that capture developmental metabolic trajectories from infancy through adolescence. These templates significantly outperformed standard adult templates in localizing epileptogenic zones in children with drug-resistant epilepsy, providing a more accurate, developmentally tailored tool for paediatric neuroimaging and preoperative evaluation.

## Graphical Abstract

**Graphical Abstract:**
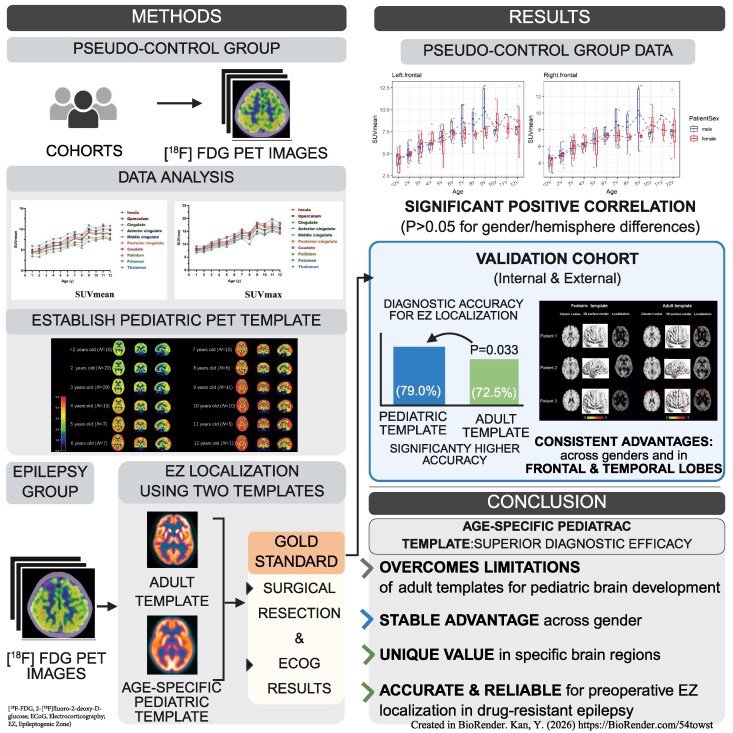

## Introduction

The paediatric period is a critical window for brain development, during which the structure and function of the brain undergo dramatic and sophisticated changes that lay the foundation for lifelong cognitive, emotional and behavioural abilities. Brain glucose metabolism, as a direct reflection of cerebral energy metabolism and neural activity, is closely associated with the maturation of neural circuits, synaptic pruning and myelination processes.^1^ PET imaging with ^18^F-fluorodeoxyglucose (^18^F-FDG) has become a golden standard for quantifying brain glucose metabolism, providing a non-invasive approach to decipher the spatiotemporal characteristics of brain developmental trajectories at the molecular level.^2^

Accurate characterization of the developmental regularities of brain glucose metabolism across the whole paediatric age range is of great significance for distinguishing normal and abnormal brain development. Previous studies have explored metabolic patterns in specific paediatric subgroups, such as school-age children or adolescents.^3,4^ However, most of these studies have limitations including narrow age coverage and lack of systematic analysis of continuous developmental trends. This gap makes it difficult to establish a comprehensive and standardized reference framework for normal brain glucose metabolism throughout childhood, hindering the early identification of neurodevelopmental disorders characterized by abnormal metabolic patterns.

PET brain templates serve as essential tools for standardized registration, normalization and quantitative analysis of neuroimaging data. Adult brain templates have been widely used in clinical and research settings, but they are not suitable for children due to significant differences in brain volume, structure and metabolic distribution between developing and mature brains. Although several paediatric brain templates have been developed in recent years, most of them are limit to the age range of 6–18 years and suffer from insufficient validation, especially the lack of external validation using independent cohorts. This deficiency severely limits the reliability and generalizability of these templates in multi-centre studies and clinical practice, as the performance of templates may vary across different populations, imaging equipment or acquisition protocols.

To address these existing shortcomings, our study aims to (i) systematically analyse the spatiotemporal developmental regularities of brain glucose metabolism in children covering the whole paediatric age range; (ii) establish age-specific paediatric brain templates based on the metabolic characteristics of the developing brain; and (iii) conduct rigorous external validation of the established templates using independent datasets. We hypothesize that brain glucose metabolism exhibits distinct age-specific patterns during childhood, and the age-specific templates validated in this study will provide a more accurate reference for the standardization of paediatric neuroimaging analysis. The findings of this study are expected to fill the gap in the comprehensive understanding of brain metabolic development in children, refine the technical system of paediatric brain template construction and validation and provide a crucial basis for the early diagnosis and prognosis evaluation of paediatric neurodevelopmental disorders. Additionally, the standardized templates and metabolic reference data may promote the consistency of results across multi-centre studies, facilitating the advancement of paediatric neuroscience research.

## Materials and methods

### Participants

The dataset of this study included two groups, a pseudo-control group and an epilepsy group. For the pseudo-control group, we retrospectively collected data from paediatric patients who underwent pre-treatment [^18^F]FDG PET/computed tomography (CT) at the Department of Nuclear Medicine, Beijing Friendship Hospital, Capital Medical University, between January 2021 and June 2023. Following a rigorous screening process, these patients were selected to fulfil two key roles: first, to provide a foundational dataset for examining the developmental trajectories of cerebral metabolism in children, and second, to facilitate the establishment of a normative cerebral metabolism template tailored for statistical parametric mapping (SPM) analyses. The inclusion criteria were as follows: (i) age < 13 years and a medical history of <1 month; (ii) no intracranial or skull lesions and no neuropsychiatric disorders; and (iii) no use of drugs affecting cerebral metabolism (e.g. hormones, antiseizure medications) or receipt of treatments with such effects (e.g. radiotherapy, chemotherapy, immunotherapy). The exclusion criteria were (i) poor image quality or significant head movement during scanning; (ii) clinical data or pathology are incomplete; and (iii) diagnosis of epilepsy syndrome, or epileptic encephalopathy.

**Figure 1:**
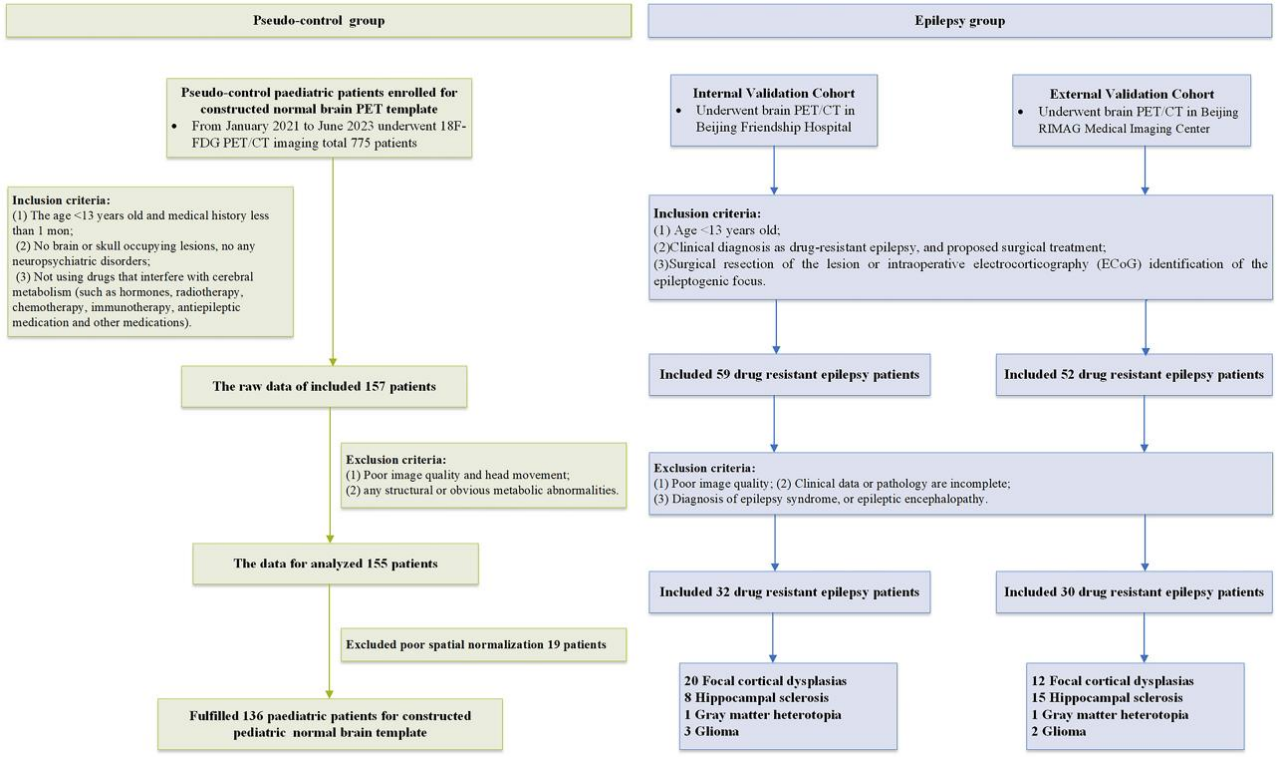
Flowchart of participant enrolment and grouping.

In addition, we retrospectively collected data from paediatric patients with drug-resistant epilepsy (DRE) who underwent pre-treatment [^18^F]FDG PET/CT between January 2021 and June 2023 at two centres: the Department of Nuclear Medicine, Beijing Friendship Hospital, Capital Medical University (the internal validation cohort), and Beijing RIMAG Medical Imaging Center (the external validation group). The inclusion criteria for this epilepsy group were (i) age < 13 years; (ii) clinically diagnosed with DRE and planned for surgical treatment; and (iii) confirmation of the epileptogenic zone (EZ) via surgical resection or intraoperative electrocorticography (ECoG). The exclusion criteria were (i) poor image quality and (ii) incomplete clinical data, insufficient pathological records, diagnosis of a generalized epilepsy and epilepsy syndrome, or diagnosis of epileptic encephalopathy (in Fig. 1).

The diagnosis of epilepsy was based on the 2014 International League Against Epilepsy practical definition,^5^ which includes the following criteria: (i) at least two unprovoked seizures occurring more than 24 h apart; (ii) one unprovoked (or reflex) seizure with a ≥60% likelihood of recurrence within the next 10 years (a risk comparable to that following two unprovoked seizures); or (iii) identification of an epilepsy syndrome. For all paediatric patients with epilepsy, the final diagnosis and localization of the primary EZ were determined based on surgical pathology, including lesion resection pathology and intraoperative stereoelectroencephalography (SEEG) with biopsy.

This study was approved by the Ethics Committee of Beijing Friendship Hospital (approval no.: BFHHZS20240072). All study procedures were performed in compliance with the Declaration of Helsinki. Every paediatric participant and their legally authorized representatives were fully briefed on the research protocol, and written informed consent was obtained before [^18^F]-FDG PET/CT imaging.

### Image acquisition and preprocessing

All [^18^F]FDG PET/CT scans were performed using a PET/CT scanner at both centres. Prior to scanning, all paediatric patients were required to fast for 4–6 h to maintain blood glucose levels between 3.9 and 6.1 mmol/L. For uncooperative children (e.g. aged <6 years), oral chloral hydrate (50 mg/kg) was administered 40 min before scanning to achieve sedation and minimize head movement. The [^18^F]FDG radiotracer was injected intravenously at a dose of 3.70 MBq/kg (0.1 mCi/kg). After injection, patients rested in a quiet, dimly lit room for 45–60 min to allow for adequate tracer uptake in the brain. CT scans were acquired first for attenuation correction and anatomical localization, followed by PET scans.

Image preprocessing was performed using SPM (SPM12, Wellcome Trust Centre for Neuroimaging, London, UK) running on MATLAB R2021a (MathWorks, Natick, MA, USA). The preprocessing pipeline included the following steps: (i) conversion of PET image formats to NIfTI for compatibility with SPM; (ii) head motion correction to eliminate artefacts caused by slight head movement during scanning; (iii) co-registration of PET images to individual T_1_-weighted MRI images to improve anatomical accuracy; and (iv) smoothing with a 5-mm full-width at half-maximum (FWHM) Gaussian kernel to reduce noise and enhance statistical power.

### The whole brain and regional brain metabolism analysis

**Figure 2:**
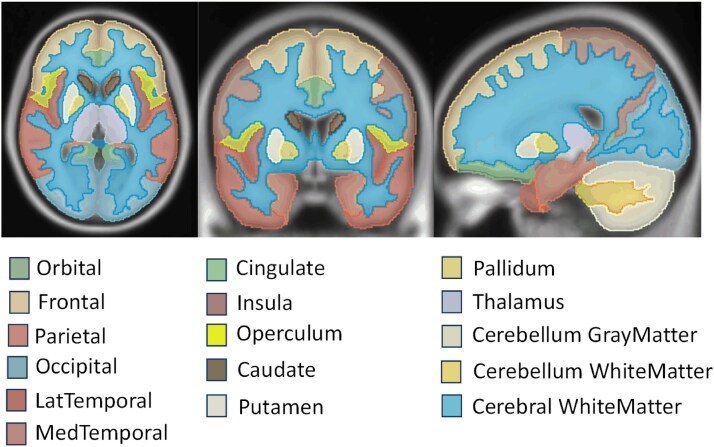
**The brain regions based on the Neuromorphometrics atlas.** We present only the axial, coronal and sagittal views of the 16 central brain regions. The merging rules for the 26 brain regions are provided in the Supplementary material.

All preprocessing brain PET images were segmented and quantified based on the Neuromorphometrics atlas, which comprises 126 distinct brain regions, including cortical and subcortical areas from both hemispheres. Due to hierarchical inclusion relationships among certain regions, we merged selected areas as needed. Ultimately, 26 brain regions across both hemispheres were included in the analysis (in Fig. 2).

From the PET images of all paediatric subjects, we extracted the maximum standardized uptake value (SUVmax), mean standardized uptake value (SUVmean) and SUV ratio (SUVR) for each region of interest (ROI) across the whole brain. We investigated the laterality of different brain regions and the variation patterns associated with the use of different reference regions for normalization. Furthermore, we examined the trends of SUVmax, SUVmean and SUVR across various brain regions in relation to age and gender. The SUVR was derived by dividing the SUVmean of each ROI by the SUVmean of a reference region. To evaluate the influence of different reference regions on data normalization, we compared the brainstem, cerebellum and the whole brain as reference areas and examined the resulting patterns of cerebral metabolism in children.

### Creation of paediatric template

**Figure 3:**
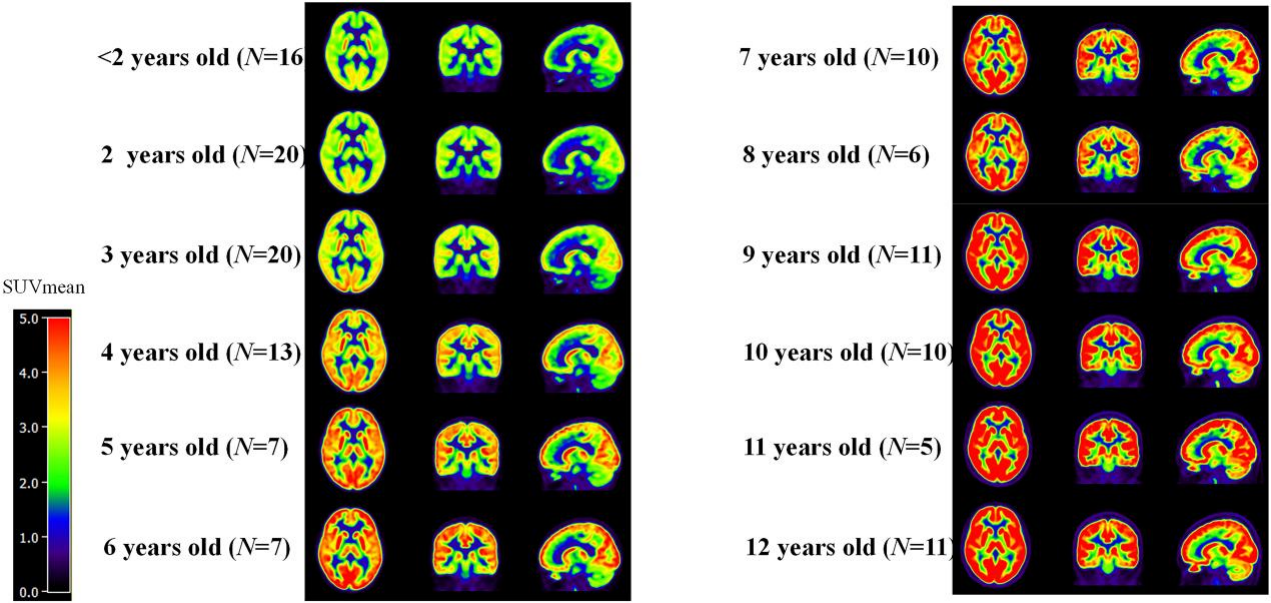
**Age-specific SPM brain templates for typically developing children.** This figure presents the sample size of participants in each age group and the corresponding [^18^F]FDG PET metabolic images of the constructed age-specific SPM brain templates.

We used the pseudo-control group to establish the paediatric template. All original [^18^F]FDG PET images were converted to SUV images utilizing paediatric weight and injection dose information. The SUV images underwent spatial normalization, including both linear and nonlinear transformations, to align with the standardized Chinese paediatric template for 6–12-year-olds.^6^ Following normalization, visual inspection was performed to exclude images with suboptimal normalization quality. Subsequently, intensity normalization was conducted using a widely adopted method, which entailed each image was divided by the mean value of pixel values within the 40th–90th percentile range in the whole brain.^7^ Mean template images were generated for each age group, and outlier images were identified using a three-standard deviation criterion.^8^ Gaussian smoothing with a FWHM of 8 mm was applied to the age-specific mean template images (Figure 3).

### The estimated epilepsy focus localization and visual inspection

For external validation of the established template, the epilepsy validation group data were used. The normalized PET images of patients with epilepsy (processed using the age-specific PET brain template and the standard adult MNI template separately) were subjected to voxel-wise statistical analysis to compare the accuracy of EZ localization. The localization of epileptic zone was performed using the DPABI toolbox (https://rfmri.org/DPABI). Cluster threshold of *P* < 0.005 (uncorrected) with a minimum extent of 50 contiguous voxels was defined as the EZ. The EZ identified by surgical resection and ECoG was used as the gold standard. The sensitivity, specificity and accuracy of EZ localization using the two templates were calculated.

### Statistical analysis

Statistical analyses were performed using SPSS 24.0 (IBM Corp., Armonk, NY, USA) and R 4.2.1 (R Foundation for Statistical Computing, Vienna, Austria). Demographic data (e.g. age, gender) of the pseudo-control group and epilepsy group were compared using independent samples *t*-tests for continuous variables. For categorical variables, the data were used *χ*² tests or one-way ANOVA. The developmental trajectories of cerebral glucose metabolism in the pseudo-control group were analysed using linear mixed-effects models, with age as the independent variable and normalized [^18^F]FDG uptake value as the dependent variable, adjusting for gender. A *P* < 0.05 was considered statistically significant for all analyses.

## Results

### Participant demographics

A rigorous screening process was conducted to select eligible participants from the retrospective cohort. A total of 136 pseudo-control subjects were finally enrolled from 775 initially recruited paediatric patients, with a screening success rate of 17.55%. These pseudo-controls were used as the foundational dataset for constructing the age-specific paediatric PET brain template. For the validation of the template, 32 paediatric drug-resistant patients with epilepsy from Beijing Friendship Hospital were enrolled as the internal validation cohort, and 30 patients from Beijing RIMAG Medical Imaging Center were included as the external validation cohort; both cohorts were used for evaluating the efficacy of EZ localization (Table 1).

**Table 1:** Demographic and clinical characteristics of patients in the pseudo-control group and the epilepsy group

Patients’ characteristics	Pseudo-control group	Epilepsy group	
		Internal validation cohort	External validation cohort
**Numbers**	136	32	30
**Gender (male/female)**	70/66	16/16	19/11
**Age (years)**	5.0 (2.3–10.0)	5.0 (3.0–8.0)	8.0 (5.0–10.0)
**Age group (*n*)**			
**1-year**	16	3	2
**2-year**	18	4	1
**3-year**	17	5	3
**4-year**	13	2	2
**5-year**	7	3	2
**6-year**	7	3	2
**7-year**	10	3	2
**8-year**	6	2	5
**9-year**	11	4	2
**10-year**	11		3
**11-year**	9	3	4
**12-year**	11		2
**Disease category**			
**Lymphoma/leukemia**	14		
**Neuroblastoma**	59		
**LCH**	3		
**Lymphoproliferative disorders**	7		
**Other malignancies**	38		
**Other benign tumors/diseases**	15		
**Causes of drug-resistant epilepsy**			
**Focal cortical dysplasias**		20	12
**Hippocampal sclerosis**		8	15
**Gr**e**y matter heterotopia**		1	1
**Glioma**		3	2

Detailed demographic characteristics of the three study groups are summarized below, with additional statistical descriptions to reflect the data distribution characteristics:

- **Pseudo-control group (*n***  **=**  **136):** The cohort consisted of 70 boys (51.47%) and 66 girls (48.53%), showing a roughly balanced gender distribution. The age range was 13 months to 12 years, with a mean age of 5.82 ± 3.21 years (mean ± standard deviation).
- **Internal validation cohort (*n***  **=**  **32):** This cohort included 16 boys (50.00%) and 16 girls (50.00%), with a perfectly balanced gender ratio. The age ranged from 14 months to 12 years, with a mean age of 6.15 ± 3.34 years.
- **External validation cohort (*n***  **=**  **30):** There were 19 boys (63.33%) and 11 girls (36.67%) in this cohort, with a slight male predominance. The age range was 12 months to 12 years, with a mean age of 7.23 ± 2.98 years, which was slightly higher than that of the internal validation cohort but without statistical significance (*P* = 0.123).

### Metabolic characteristics of different brain regions across age groups

To clarify the developmental regularities of brain glucose metabolism in normal children, we quantified the metabolic parameters (SUVmax, SUVmean, SUVR) of 26 predefined brain regions in the pseudo-control group (13 months–12 years old). Detailed SUVmean values of each brain region across different age groups are presented in Supplementary Table 1.

Correlation analysis showed that SUVmean and SUVmax in all 26 brain regions were significantly positively correlated with age (*r* = 0.32–0.61, *P* < 0.001), indicating a gradual increase in brain glucose metabolism with age, which is consistent with the continuous maturation process of the paediatric brain.

Gender-based stratification analysis showed no statistically significant differences in SUVmean or SUVmax between boys and girls in the same age group (*P* > 0.05; Supplementary Figs 1 and 2), indicating that gender has little impact on brain glucose metabolism in children. In addition, comparison of metabolic parameters between the left and right hemispheres of the same brain region also showed no significant differences (*P* > 0.05; Supplementary Figs 3 and 4), reflecting the symmetry of normal paediatric brain glucose metabolism.

**Figure 4:**
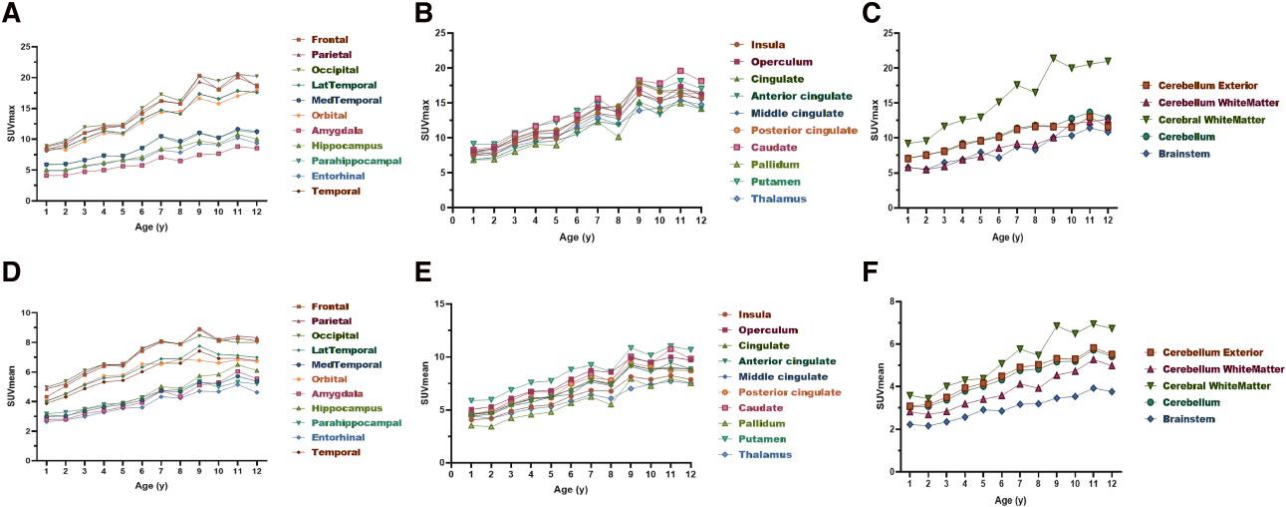
**Age-related trends of SUVmax and SUVmean across 26 brain regions.** This figure demonstrates that SUVmax (**A–C**) and SUVmean (**D–F**) values in the 26 predefined brain regions exhibited a general increasing trend with advancing chronological age. The participants were stratified into single-year age groups with the following sample sizes: 1 year (*N* = 16), 2 years (*N* = 18), 3 years (*N* = 17), 4 years (*N* = 13), 5 years (*N* = 7), 6 years (*N* = 7), 7 years (*N* = 10), 8 years (*N* = 6), 9 years (*N* = 11), 10 years (*N* = 11), 11 years (*N* = 9) and 12 years (*N* = 11). Each coordinate point on the trend line represents the mean value of all participants in the corresponding single-year age group. SUVmax, maximum standardized uptake value; SUVmean, mean standardized uptake value.

**Figure 5:**
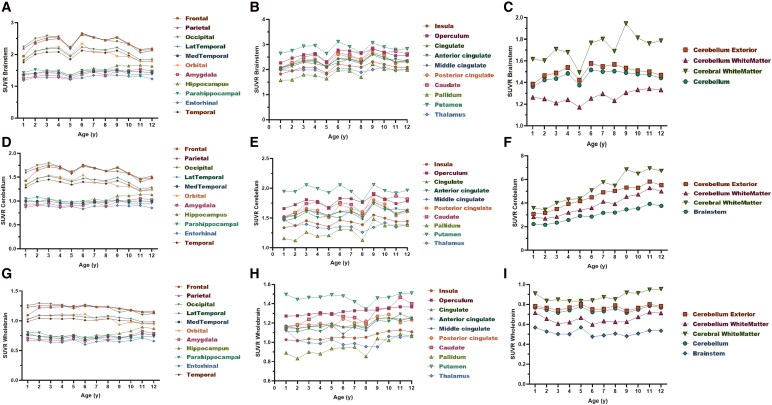
**Age-related trend of SUVR across 26 brain regions.** This figure shows a consistent increasing trend in SUVR values across the 26 predefined brain regions, including the brainstem (**A–C**), cerebellum (**D–F**) and whole brain (**G–I**), with increasing age. The participants were stratified into single-year age groups with the following sample sizes: 1 year (*N* = 16), 2 years (*N* = 18), 3 years (*N* = 17), 4 years (*N* = 13), 5 years (*N* = 7), 6 years (*N* = 7), 7 years (*N* = 10), 8 years (*N* = 6), 9 years (*N* = 11), 10 years (*N* = 11), 11 years (*N* = 9) and 12 years (*N* = 11). Each coordinate point on the trend line represents the mean value of all participants in the corresponding single-year age group. SUVR, standardized uptake value ratio.

The age-related trends of SUVmax and SUVR in each brain region were consistent with those of SUVmean, further confirming the reliability of the metabolic development trajectory (Figs 4 and 5). For the normalization method, we compared three commonly used references (cerebellum, brainstem, whole brain) and found no statistically significant differences in the calculated metabolic parameters (*P* > 0.05; Supplementary Table 2), indicating that the choice of normalization reference has little impact on the results of paediatric brain metabolic analysis in this study.

### Diagnostic performance of paediatric versus adult templates

**Figure 6:**
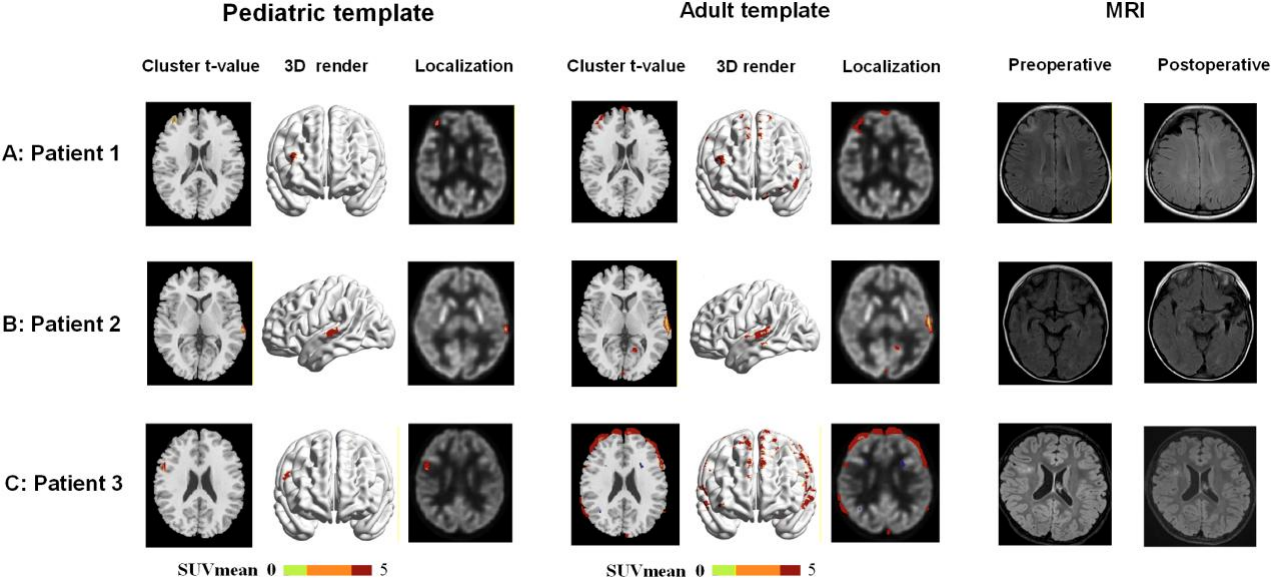
**Comparison of EZ localization accuracy between paediatric age-specific and adult templates in three paediatric DRE patients with focal cortical dysplasia (FCD).** (**A**) Patient 1: An 8-year-old girl admitted for intermittent epilepsy refractory to antiepileptic drug (AED) therapy for over 3 years. [^18^F]FDG PET revealed FDG hypometabolism in the anterior frontal gyrus and adjacent superior frontal gyrus of the right frontal lobe, consistent with interictal epileptic manifestations. The patient underwent curative right frontal EZ resection, with postoperative pathology confirming FCD type IIa and 12-month seizure freedom (Engel Class I). (**B**) Patient 2: 2-year-old boy with DRE (1-year disease course, multifocal parieto-occipitotemporal seizures). [^18^F]FDG PET showed multifocal hypometabolism in the left parietal, occipital and temporal lobes, compatible with interictal epileptic manifestations. Left temporal EZ resection was performed, with postoperative pathology verifying FCD type IIb. (**C**) Patient 3: 5-year-old female with DRE (2-year disease course, frontal seizures). [^18^F]FDG PET demonstrated mild focal hypermetabolism in the right frontal cortical and subcortical regions, suggestive of ictal metabolic alterations. The patient underwent stereotactic electroencephalography (SEEG) and radiofrequency ablation, with pathological confirmation of FCD type I in the right frontal EZ and 6-month seizure reduction (Engel Class II).

We compared the diagnostic efficacy of the constructed age-specific paediatric SPM template and the standard adult SPM template in EZ localization using [^18^F]FDG PET images of drug-resistant patients with epilepsy (2–12 years old) from the internal and external validation cohorts. The EZs (32 in the internal cohort, 30 in the external cohort) areas were hypometabolic upon visual analysis, which is consistent with the typical metabolic manifestation of DRE (Fig. 6).

### Internal validation cohort

The overall diagnostic accuracy of the paediatric template in EZ localization was 81.3% (26/32), slightly higher than that of the adult SPM template (78.1%, 25/32), but the difference was not statistically significant (*P* = 0.721).

**Subgroup analysis by brain region revealed distinct performance characteristics of the two templates across different anatomical sites:**

- Parietal lobe (*n* = 2): Both templates showed moderate and comparable accuracy (50.0% for each), which may be related to the small sample size of this subgroup.
- Frontal lobe (*n* = 18): The paediatric SPM template showed significantly higher accuracy than adult template (88.9% versus 50.0%, *P* = 0.012).
- Temporal lobe (*n* = 11): The paediatric template also outperformed the adult template (72.7% versus 45.5%), although the difference was not statistically significant (*P* = 0.186) due to the limited sample size.
- Cingulate gyrus (*n* = 1): Both templates and visual assessment achieved 100% accuracy (95%), indicating good performance in localizing rare EZ sites.

**Gender-stratified analysis further showed differences in template performance between boys and girls:**

- Boys (*n* = 16): The paediatric template achieved an accuracy of 87.5% (14/16), whereas the adult template reached 68.8% (11/16). Statistical comparison confirmed that the diagnostic accuracy of the paediatric template was significantly superior to that of the adult template (*P* = 0.043).
- Girls (*n* = 16): The paediatric SPM template exhibited an accuracy of 68.8% (11/16), while the adult SPM template showed the lowest accuracy (31.2%, 5/16). A statistically significant difference in diagnostic accuracy was observed between the two templates (*P* = 0.021) (Table 2).

**Table 2:** Comparison of the diagnostic accuracy of the paediatric-specific template with the adult template for detecting drug-resistant epileptic foci across different age subgroups in the epilepsy group

Age	Number	Internal validation cohort(*n* = 32)			External validation cohort(*n* = 30)		
		*N*	Paediatric template (*n*, %)	Adult template (*n*, %)	*N*	Paediatric template (*n*, %)	Adult template (*n*, %)
**<2-year**	5	3	3/3 100.0%	3/3 100.0%	2	2/2 100.0%	2/2 100.0%
**2-year**	5	4	4/4 100.0%	4/4 100.0%	1	0/1 0.0%	0/1 0.0%
**3-year**	6	5	4/5 80.0%	3/5 60.0%	1	1/1 100.0%	1/1 100.0%
**4-year**	4	2	1/2 50.0%	1/2 50.0%	2	2/2 100.0%	2/2 100.0%
**5-year**	5	3	2/3 66.7%	2/3 66.7%	2	2/2 100.0%	1/2 50.0%
**6-year**	5	3	2/3 66.7%	2/3 66.7%	2	1/2 50.0%	0/2 0.0%
**7-year**	5	3	2/3 66.7%	3/3 100.0%	2	1/2 50.0%	1/2 50.0%
**8-year**	8	2	2/2 100.0%	2/2 100.0%	6	6/6 100.0%	6/6 100.0%
**9-year**	6	4	4/4 100.0%	4/4 100.0%	2	1/2 50.0%	2/2 100.0%
**10-year**	4				4	3/4 75.0%	3/4 75.0%
**11-year**	7	3	2/3 66.7%	1/3 33.3%	4	2/4 50.0%	1/4 25.0%
**12-year**	2				2	2/2 100.0%	2/2 100.0%
**Total**	62	32	26/32 81.3%	25/32 78.1%	30	23/30 76.7%	21/30 70.0%

### External validation cohort

Consistent with the internal validation results, the overall diagnostic accuracy of the paediatric SPM template was 76.7% (23/30), which was identical to that of the adult SPM template (70.0%, 21/30; *P* = 0.374). Brain region-specific subgroup analysis showed trends similar to the internal cohort, with several notable variations:

- Parietal lobe (*n* = 4): The paediatric SPM template achieved all, while the adult template had an accuracy of 75.0%. No statistically significant difference was observed between the two templates (*P* = 0.444).
- Frontal lobe (*n* = 9): The paediatric template showed higher accuracy (66.7%) than the adult template (44.4%), consistent with the internal validation results.
- Temporal lobe (*n* = 16): Both templates exhibited identical accuracy (56.3%), indicating consistent but relatively limited performance in this brain region compared to the gold standard.
- Occipital lobe (*n* = 1): The paediatric template correctly localized all the EZ, while the adult template failed. This finding suggests that the paediatric template may have unique advantages in localizing EZs in brain regions with significant developmental differences between children and adults (Table 2).

### Integrated analysis of validation results

Combining the results of internal and external validation cohorts (total *n* = 62 patients with epilepsy), the overall diagnostic accuracy of the self-established paediatric SPM template was 79.0%, which was significantly higher than that of the standard adult SPM template 72.5% (*P* = 0.033). This integrated analysis further confirmed the superiority of the paediatric template in EZ localization for paediatric DRE, with key findings refined as follows.

First, in terms of overall diagnostic efficacy, the paediatric SPM template achieved superior performance to the adult template. Specifically, the marginal advantage in overall accuracy observed in the integrated cohort reached statistical significance, which was supported by consistent trends in both internal and external validation subsets, confirming the stable and reliable performance of the paediatric template across independent populations.

Second, the updated gender-stratified analysis (integrating internal validation cohort data) revealed a consistent advantage of the paediatric SPM template over the adult template across sex, which differed from the previously observed gender-specific discrepancy. This finding indicated that the diagnostic advantage of the paediatric template is not affected by gender, providing a more consistent normative reference for EZ localization in both boys and girls with DRE.

Third, the brain region-specific performance pattern was further validated in the integrated analysis: the paediatric template showed better performance in frontal and temporal lobe EZ localization. Notably, the paediatric template’s advantage in gender consistency complemented its brain region-specific strengths, making it more adaptable to the heterogeneous anatomical and developmental characteristics of paediatric epilepsy.

Collectively, the integrated analysis of internal and external validation data demonstrated that the paediatric SPM template not only achieves overall superior diagnostic efficacy for EZ localization in paediatric DRE but also maintains stable advantages across sex and exhibits unique value in specific brain regions. These findings fully support the clinical applicability of the paediatric template, as it addresses the limitations of adult templates in adapting to paediatric brain development and gender-related metabolic differences, providing a more accurate and reliable tool for preoperative EZ localization in paediatric DRE.

## Discussion

Our study first characterized the age-related increasing trends in cerebral metabolism across 26 brain regions in children and confirmed that no gender-related differences in metabolic parameters were observed. Subsequently, based on these findings, we developed a paediatric-specific PET template for paediatric neuroimaging research and validated its utility in DRE among paediatric patients. When compared to the commonly used adult template, the paediatric PET template exhibited comparable diagnostic accuracy for epilepsy. Thereby, it potentially enables more precise localization of EZs.

Accurate identification of the EZ is critical for epilepsy management, regardless of whether the EZ presents as a distinct anatomical lesion or a functional abnormality.^9^ The application of objective semi-quantitative ROI techniques or voxel-based SPM has been validated as valuable for preoperative EZ localization.^10,11^ Notably, SPM analysis can detect subtle metabolic abnormalities that may be overlooked in conventional visual assessments.^9,12^ A standardized brain template derived from healthy controls is indispensable for accurate EZ localization using SPM; however, most existing templates are constructed based on normal adult brains. Owing to the rapid developmental changes in cerebral metabolism throughout childhood and significant morphological disparities between paediatric and adult brains, the use of adult SPM templates compromises the efficacy of diagnosis and management of paediatric neurological disorders.^13-15^ Prior research has further indicated that employing adult templates and standardized adult procedures may introduce substantial inaccuracies in SPM analysis for children under 6 years of age.^16^ Consequently, constructing age-appropriate normal brain SPM templates is essential for diagnosing paediatric brain diseases.

Understanding the typical patterns of [^18^F]FDG uptake in paediatric brains is a prerequisite for developing normal SPM templates, which in turn facilitates the identification of pathological metabolic changes associated with paediatric neurological disorders.^17^ The previous studies have demonstrated that the cerebral FDG uptake patterns undergo continuous alterations with advancing age.^17,18^ Furthermore, our findings revealed a consistent increasing trend of [^18^F]FDG uptake across various cerebral regions with age, and this further justifies the need for age-dependent paediatric templates, as was done in this study. In contrast to these findings, Barber *et al*. studied 28 subjects with normal cerebral metabolism (11 children and 17 adolescents) and observed asymmetric SUVmean values between bilateral brain regions.^19^ In our study, we found that the upward trend of bilateral brains is similar in different age groups. Our results indicated that the upward metabolic trend was consistent between bilateral brain regions across different age groups. Importantly, our data suggested that even minor age differences can lead to discernible variations in cerebral metabolic activity in children. Thus, constructing normal brain templates tailored to specific paediatric age groups can more accurately support the diagnosis of paediatric neurological diseases.

Due to ethical concerns, developing a fully standardized normal brain SPM template exclusively for children is impractical. Therefore, age-specific templates of cerebral metabolism in normal children were constructed using ‘pseudo-normal’ paediatric subjects, including patients with normal cerebral metabolism, deaf children or paediatric patients with extracranial malignant tumours without cerebral involvement.^9,20,21^ Notably, prior studies on cerebral metabolism in ‘pseudo-control’ children have consistently demonstrated a gradual increase in [^18^F]FDG uptake with age, while revealing heterogeneous growth rates of cerebral metabolism across different cortical regions.^15,17,18^ Our study primarily focused on metabolic analysis of various brain regions in a cohort of ‘pseudo-normal’ children aged 13 months to 12 years. The results indicated a positive correlation between age and glucose metabolic parameters across all brain regions. Importantly, compared to previous studies, we further subdivided the brain into 26 distinct regions and utilized the Neuromorphometrics atlas for precise delineation, thereby enhancing the accuracy of analytical results and laying a solid foundation for developing more precise paediatric SPM templates.

Given the dynamic changes in cerebral volume and metabolism during childhood, employing adult SPM templates for paediatric EZ diagnosis may introduce bias; in contrast, age-specific brain templates can minimize standardization errors in paediatric cerebral [^18^F]FDG PET images.^21^ Several prior studies have validated the efficacy of constructing age-specific normal paediatric brain templates for accurate diagnosis of paediatric brain diseases.^20-24^ For instance, De Blasi *et al*. divided 112 subjects (6–20 years old) into two groups using a 10-year-old cut-off (19 subjects aged 6–9 years and 93 subjects aged 10–20 years) and constructed age-specific normal paediatric brain templates,^22^ providing an accurate and sensitive semi-quantitative method for diagnosing patients under 18 years of age. While this approach proved valuable, their study lacked more granular age stratification. Zhu *et al*. further suggested that for SPM analysis of cerebral [^18^F]FDG PET images, age-matched controls should be stratified into as many subgroups as possible, proposing age groups of 1 year, 2–3 years, 4–6 years, 7–10 years, 11–14 years and 15–18 years.^21^ To our knowledge, our study is the first to construct age-specific paediatric brain templates for each year of age ranging from 2 to 12 years, thus filling a critical gap in existing literature, followed by a comparison with conventional adult SPM templates in diagnosing paediatric DRE. We found that adult templates were more susceptible to false-positive non-target EZs compared to paediatric templates, which may interfere with the localization of primary EZs (Fig. 6). This discrepancy may be attributed to the overall lower cerebral metabolism in children relative to adults. Despite this limitation, both templates achieved favourable diagnostic performance.

This study has several limitations. First, the sample size of the integrated cohort remains relatively small (total *n* = 62). Although both internal and external validation cohorts were included to enhance the reliability of the results, the limited number of cases may restrict the statistical power to detect subtle differences in diagnostic efficacy, especially for rare subtypes of paediatric DRE or EZ located in less common brain regions (e.g. occipital lobe, parietal lobe). This may also affect the generalizability of the findings to larger and more diverse paediatric populations. Second, the study population may have inherent selection bias. The included patients were likely from specific clinical centres, which may have particular characteristics of epilepsy aetiology and may limit the applicability of the paediatric SPM template to more complex clinical scenarios. Third, the current study focused on the diagnostic accuracy of EZ localization based on imaging data but lacked long-term follow-up data to evaluate the clinical impact of the paediatric template. This limitation will be supplemented in subsequent studies by larger cohorts to further validate the generalizability of our findings and conducting long-term follow-up of enrolled patients to collect surgical outcome data and clarify the clinical value of the paediatric SPM template in improving patient prognosis.

## Conclusion

Our study characterized the developmental trajectories of cerebral metabolism across various brain regions in paediatric populations. Collectively, the constructed age-specific paediatric SPM template exhibits superior EZ localization efficacy to the traditional adult SPM template and generates fewer false-positive EZs. Therefore, the construction of paediatric-specific SPM templates is warranted to improve the accuracy of EZ localization in children with DRE, with potential implications for optimizing preoperative evaluation and clinical outcomes.

## Supplementary Material

fcag280_Supplementary_Data

## Data Availability

Original data of this study are available from the corresponding authors upon reasonable request. All analysis codes are provided in the supplementary materials of this article.
