# Supplementary material for: Development of an age-specific paediatric 2-[18F]fluoro-2-deoxy-D-glucose PET template aligned with paediatric brain development regularities: enhancing epileptogenic zone localization in drug-resistant epilepsy
Source: Brain Commun. 2026 Jul 16;8(4):fcag280. doi: 10.1093/braincomms/fcag280 (PMC13399154; doi:10.1093/braincomms/fcag280)
Supplement: fcag280_Supplementary_Data [file fcag280_supplementary_data.pdf]

Supplementary Table 1. The mean standardized uptake value (SUVmean) values of different brain areas in different age group.

Age (Years)	Orbital	Frontal	Parietal	Occipital	LatTemporal	MedTemporal
**1**	8.44<br>(6.76, 10.12)	8.21<br>(7.33, 9.09)	8.61<br>(7.63, 9.59)	8.74<br>(7.59, 9.87)	7.96<br>(6.96, 8.96)	5.81<br>(5.15, 6.48)
**2**	8.28<br>(7.58, 8.98)	9.12<br>(8.49, 9.74)	9.52<br>(8.82, 10.22)	9.75<br>(8.87, 10.63)	8.74<br>(8.02, 9.47)	5.98<br>(5.59, 6.37)
**3**	9.63<br>(8.88, 10.40)	11.02<br>(10.08, 11.98)	11.03<br>(10.08, 11.96)	11.96<br>(10.71, 13.19)	10.15<br>(9.35, 10.96)	6.61<br>(6.01, 7.12)
**4**	10.98<br>(9.97, 12.00)	11.81<br>(10.62, 12.98)	12.08<br>(10.84, 13.32)	12.29<br>(10.70, 13.87)	11.33<br>(10.38, 12.28)	7.33<br>(6.57, 8.10)
**5**	10.81<br>(7.61, 14.02)	12.15<br>(8.95, 15.35)	12.09<br>(8.95, 15.23)	12.37<br>(8.49, 16.24)	11.01<br>(8.22, 13.79)	7.28<br>(5.47, 9.10)
**6**	12.76<br>(10.82, 14.71)	14.11<br>(12.02, 16.20)	14.56<br>(11.98, 17.15)	15.06<br>(12.28, 17.83)	13.28<br>(11.01, 15.55)	8.54<br>(6.73, 10.37)
**7**	14.38<br>(11.65, 17.10)	16.19<br>(12.81, 19.57)	16.23<br>(13.28, 19.26)	17.26<br>(13.45, 21.06)	14.70<br>(12.16, 17.24)	10.49<br>(8,12, 12.87)
**8**	14.52<br>(11.04, 18.00)	15.78<br>(10.46, 21.01)	15.79<br>(11.19, 20.39)	16.24<br>(10.73, 21.78)	14.12<br>(10.47, 17.78)	9.72<br>(6.47, 12.97)
**9**	16.62<br>(12.66, 20.58)	20.27<br>(14.82, 25.73)	19.33<br>(14.97, 23.70)	20.27<br>(15.11, 25.43)	17.35<br>(13.05, 21.65)	11.07<br>(8.89, 13.24)
**10**	15.78<br>(13.26, 18.31)	18.05<br>(15.08, 21.02)	18.22<br>(15.22, 21.21)	19.47<br>(15.62, 23.33)	16.56<br>(13.27, 19.86)	10.23<br>(8.32, 12.12)
**11**	17.01<br>(13.03, 20.98)	20.02<br>(14.69, 25.35)	20.63<br>(14.75, 26.51)	20.54<br>(13.66, 27.42)	17.84<br>(13.28, 22.40)	11.64<br>(8.11, 15.17)
**12**	17.96<br>(14.82, 21.09)	18.74<br>(16.49, 20.99)	18.57<br>(16.18, 20.97)	20.20<br>(17.42, 22.98)	17.61<br>(15.41, 19.81)	11.25<br>(9.76, 12.75)

(Mean, 95%CI)

Supplementary Table 1. The SUVmean values of different brain areas in different age group. (Continued)

Age (Years)	Amygdala	Hippocampus	Parahippocampus	Enrorhinal	Temporal	Insula
**1**	4.09<br>(3.63, 4.54)	4.93<br>(4.49, 5.37)	5.78<br>(5.11, 6.45)	4.83<br>(4.38, 5.27)	8.11<br>(7.08, 9.13)	7.47<br>(6.59, 8.34)
**2**	4.14<br>(3.92, 4.35)	5.02<br>(4.77, 5.27)	5.95<br>(5.55, 6.35)	4.91<br>(4.60, 5.23)	8.74<br>(8.02, 9.47)	7.64<br>(6.97, 8.31)
**3**	4.74<br>(4.40, 5.07)	5.71<br>(5.27, 6.13)	6.59<br>(6.07, 7.11)	5.62<br>(5.16, 6.09)	10.15<br>(9.35, 10.95)	9.18<br>(8.39, 9.98)
**4**	5.00<br>(4.59, 5.41)	6.20<br>(5.63, 6.78)	7.33<br>(6.57, 8.10)	5.98<br>(5.37, 6.59)	11.33<br>(10.38, 12.28)	10.00<br>(9.00, 11.01)
**5**	5.63<br>(4.25, 7.01)	6.60<br>(4.98, 8.21)	7.28<br>(5.46, 9.10)	6.64<br>(4.89, 8.39)	11.01<br>(8.22, 13.79)	9.93<br>(7.18, 12.68)
**6**	5.75<br>(4.71, 6.79)	7.14<br>(5.77, 8.51)	8.55<br>(6.73, 10.37)	6.75<br>(5.66, 7.83)	13.28<br>(11.01, 15.54)	12.30<br>(10.89, 13.71)
**7**	7.05<br>(5.63, 8.47)	8.43<br>(6.91, 9.95)	10.44<br>(8.03, 12.85)	8.27<br>(6.75, 9.79)	14.70<br>(12.16, 17.24)	13.54<br>(10.91, 16.18)
**8**	6.50<br>(4.84, 8.17)	8.78<br>(5.39, 12.16)	9.37<br>(6.73, 12.02)	7.87<br>(5.20, 10.54)	14.12<br>(10.47, 17.77)	13.10<br>(8.85, 17.35)
**9**	7.47<br>(6.14, 8.79)	9.73<br>(7.98, 11.48)	10.92<br>(8.71, 13.13)	9.28<br>(7.48,11.08)	17.35<br>(13.05, 21.64)	16.26<br>(11.56, 20.95)
**10**	7.66<br>(6.23, 9.09)	9.26<br>(7.52, 11.00)	10.23<br>(8.33, 12.12)	9.07<br>(7.20, 10.95)	16.57<br>(13.26, 19.86)	15.38<br>(12.11, 18.65)
**11**	8.82<br>(6.51, 11.13)	10.79<br>(7.34, 14.24)	11.40<br>(8.23, 14.56)	10.23<br>(7.66, 12.81)	17.84<br>(13.28, 22.40)	16.03<br>(12.98, 19.09)
**12**	8.54<br>(7.67, 9.39)	10.01<br>(9.12, 10.91)	11.09<br>(9.58, 12.59)	9.37<br>(8.35, 10.39)	17.61<br>(15.42, 19.80)	15.66<br>(13.97, 17.35)

(Mean, 95%CI)

Supplementary Table 1. The SUVmean values of different brain areas in different age group. (Continued)

Age (Years)	Operculum	Cingulate	Anterior Cingulate	Middle Cingulate	Posterior Cingulate	Caudate
**1**	8.07<br>(7.15, 8.98)	7.88<br>(7.01, 8.75)	6.93<br>(6.20, 7.66)	7.56<br>(6.76, 8.36)	7.68<br>(6.81, 8.55)	8.27<br>(7.31, 9.24)
**2**	8.45<br>(7.72, 9.19)	8.54<br>(7.84, 9.23)	7.16<br>(6.66, 7.66)	7.94<br>(7.35, 8.54)	8.41<br>(7.67, 9.14)	8.70<br>(8.10, 9.30)
**3**	9.58<br>(8.82, 10.35)	9.97<br>(9.07, 10.88)	8.47<br>(7.83, 9.08)	9.24<br>(8.48, 9.99)	9.91<br>(9.01, 10.81)	10.49<br>(9.60, 11.37)
**4**	10.57<br>(9.68, 11.47)	10.97<br>(9.82, 12.11)	9.31<br>(8.39, 10.23)	10.17<br>(9.21, 11.10)	10.80<br>(9.67, 11.93)	11.64<br>(10.43, 12.84)
**5**	10.70<br>(7.89, 13.51)	11.09<br>(7.83, 14.35)	9.61<br>(6.89, 12.32)	10.04<br>(7.39, 12.67)	11.09<br>(7.83, 14.35)	12.71<br>(9.04, 16.39)
**6**	12.70<br>(10.84, 14.57)	12.50<br>(10.17, 14.83)	10.53<br>(8.96, 12.11)	11.75<br>(9.78, 13.71)	12.50<br>(10.17, 14.83)	13.28<br>(11.59, 14.96)
**7**	14.44<br>(11.78, 17.09)	14.29<br>(11.39, 17.21)	12.36<br>(9.67, 15.06)	13.39<br>(10.59, 16.18)	14.23<br>(11.29, 17.17)	15.61<br>(12.50, 18.72)
**8**	13.78<br>(9.86, 17.61)	14.56<br>(9.33, 19.78)	11.89<br>(7.67, 16.11)	12.75<br>(8.55, 16.95)	14.53<br>(9.27, 19.78)	13.69<br>(9.39, 18.01)
**9**	16.95<br>(12.58, 21.31)	17.95<br>(13.17, 22.72)	14.76<br>(11.35, 18.17)	16.25<br>(12.75, 19.75)	17.67<br>(12.90, 22.46)	18.22<br>(13.46, 22.98)
**10**	15.47<br>(12.85, 18.09)	16.73<br>(13.94, 19.51)	13.31<br>(10.54, 16.07)	15.08<br>(12.55, 17.61)	16.62<br>(13.79, 19.44)	17.81<br>(14.62, 20.99)
**11**	17.34<br>(13.01, 21.68)	16.84<br>(13.56, 20.12)	15.64<br>(12.22, 19.06)	16.57<br>(13.51, 19.63)	16.44<br>(12.90, 19.98)	19.60<br>(13.51, 25.69)
**12**	16.15<br>(14.18, 18.12)	16.57<br>(14.93, 18.20)	14.15<br>(12.46, 15.83)	15.49<br>(13.47, 17.46)	16.31<br>(14.80, 17.82)	18.12<br>(16.12, 20.14)

(Mean, 95%CI)

Supplementary Table 1. The SUVmean values of different brain areas in different age group. (Continued)

Age (Years)	Pallidum	Putamen	Thalamus	Cerebellum	Cerebellum Exterior
**1**	6.84<br>(5.87, 7.81)	9.13<br>(7.95, 10.29)	7.97<br>(6.95, 8.97)	7.09<br>(6.21, 7.99)	7.06<br>(6.16, 7.97)
**2**	6.92<br>(6.31, 7.54)	9.08<br>(8.36, 9.80)	7.68<br>(7.18, 8.18)	7.60<br>(6.88, 8.32)	7.55<br>(6.80, 8.29)
**3**	8.01<br>(7.28, 8.74)	10.68<br>(9.79, 11.56)	8.89<br>(8.15, 9.63)	8.14<br>(7.23, 9.04)	8.11<br>(7.18, 9.04)
**4**	9.07<br>(8.16, 9.98)	11.70<br>(10.46, 12.95)	9.51<br>(8.61, 10.42)	9.20<br>(7.99, 10.41)	8.98<br>(7.76, 10.20)
**5**	8.95<br>(6.19, 11.70)	12.28<br>(9.18, 15.36)	10.34<br>(7.83, 12.86)	9.66<br>(7.57, 11.76)	9.59<br>(7.44, 11.75)
**6**	11.14<br>(9.58, 12.71)	13.87<br>(11.85, 15.88)	11.36<br>(9.76, 12.96)	10.31<br>(8.42, 12.19)	10.11<br>(8.08, 12.15)
**7**	12.28<br>(9.72, 14.85)	14.73<br>(11.61, 17.86)	12.88<br>(10.72, 15.05)	11.40<br>(8.69, 14.11)	11.23<br>(8.46, 14.02)
**8**	10.13<br>(6.80, 13.46)	13.55<br>(9.08, 18.01)	11.98<br>(8.49, 15.46)	11.79<br>(8.94, 14.65)	11.63<br>(8.48, 14.77)
**9**	15.14<br>(10.63, 19.67)	17.92<br>(13.28, 22.56)	13.92<br>(10.99, 16.85)	11.71<br>(9.23, 14.18)	11.62<br>(9.09, 14.15)
**10**	14.01<br>(11.26, 16.78)	17.02<br>(14.49, 19.54)	14.29<br>(11.62, 16.96)	12.77<br>(10.63, 14.91)	11.53<br>(9.37, 13.68)
**11**	14.95<br>(10.38, 19.52)	18.15<br>(13.03, 23.28)	15.32<br>(10.93, 19.71)	13.67<br>(9.42, 17.91)	13.01<br>(9.10, 16.93)
**12**	14.15<br>(12.02, 16.28)	17.04<br>(14.95, 19.15)	14.77<br>(13.10, 16.44)	12.86<br>(11.81, 13.91)	11.60<br>(10.58, 12.62)

(Mean, 95%CI)

Supplementary Table 2. The SUVR values of three different reference regions in different age groups.

Age (Years)	Cerebellum White matter	Cerebral White matter	Brain Stem
**1**	5.79 (5.15, 6.43)	9.21 (8.06, 10.37)	5.78 (5.18, 6.39)
**2**	5.47 (5.14, 5.81)	9.57 (8.88, 10.27)	5.51 (5.15, 5.88)
**3**	5.93 (5.54, 6.31)	11.67 (10.55, 12.79)	6.48 (6.04, 6.91)
**4**	6.89 (6.04, 7.75)	12.57 (10.87, 14.26)	6.90 (6.08, 7.73)
**5**	7.32 (5.61, 9.05)	12.95 (9.20, 16.71)	7.95 (6.56, 9.34)
**6**	8.56 (7.08, 10.04)	15.14 (12.79, 17.49)	7.16 (6.09, 8.23)
**7**	9.16 (7.61, 10.72)	17.58 (13.69, 21.48)	8.75 (7.16, 10.34)
**8**	9.05 (6.72, 11.37)	16.51 (11.22, 21.81)	8.32 (6.11, 10.52)
**9**	10.05 (8.63, 11.48)	21.39 (15.46, 27.33)	10.08 (8.21, 11.94)
**10**	12.04 (9.98, 14.10)	19.99 (16.04, 23.94)	10.34 (8.78, 11.89)
**11**	12.34 (8.33, 16.36)	20.54 (14.91, 26.17)	11.44 (8.25, 14.63)
**12**	12.69 (11.69, 13.69)	20.98 (18.23, 23.74)	10.85 (9.56, 12.14)

(Mean, 95%CI)

Supplementary Figure 1: Differences in the changing trends of SUVmean values across various brain regions between genders (Two independent-samples t-test were performed for all data presented in Fig A–Z. The *T*-values ranged from −1.56 to 2.308, and *P*-values ranged from 0.104 to 0.989; all *P*-values were greater than 0.05. Each datapoint represents data from an independent single sample, and each boxplot presents the mean ± standard deviation of samples in each age group).

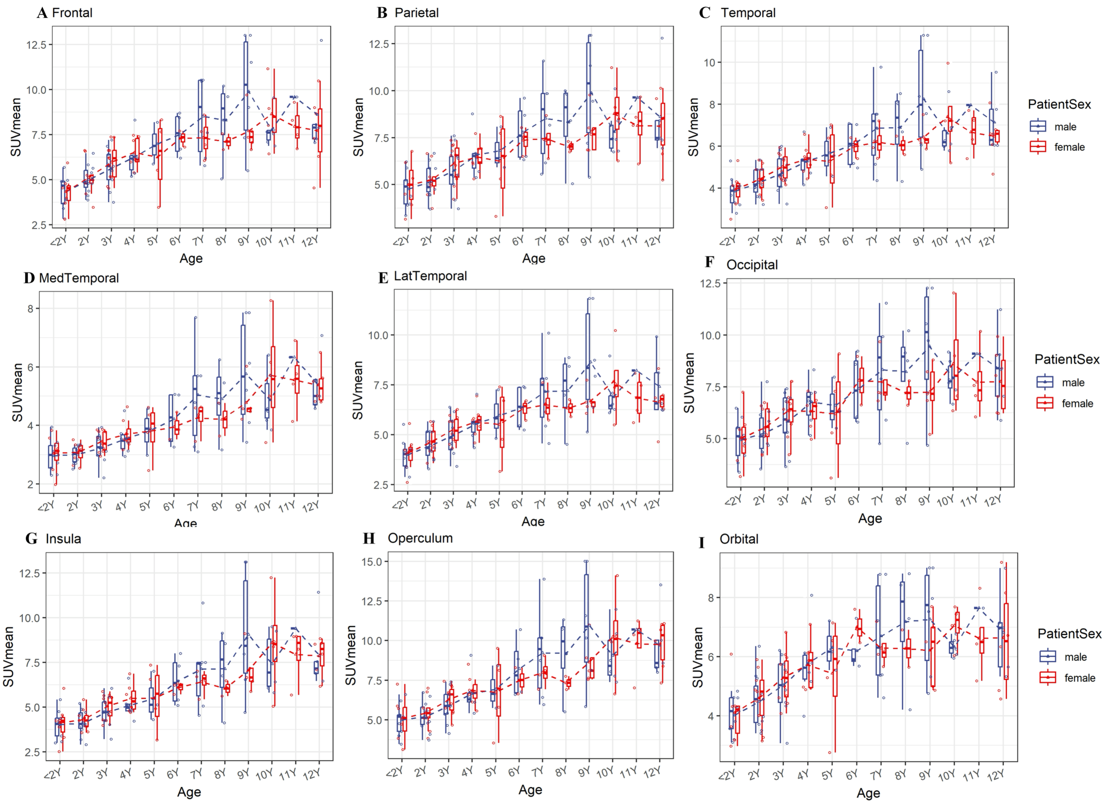

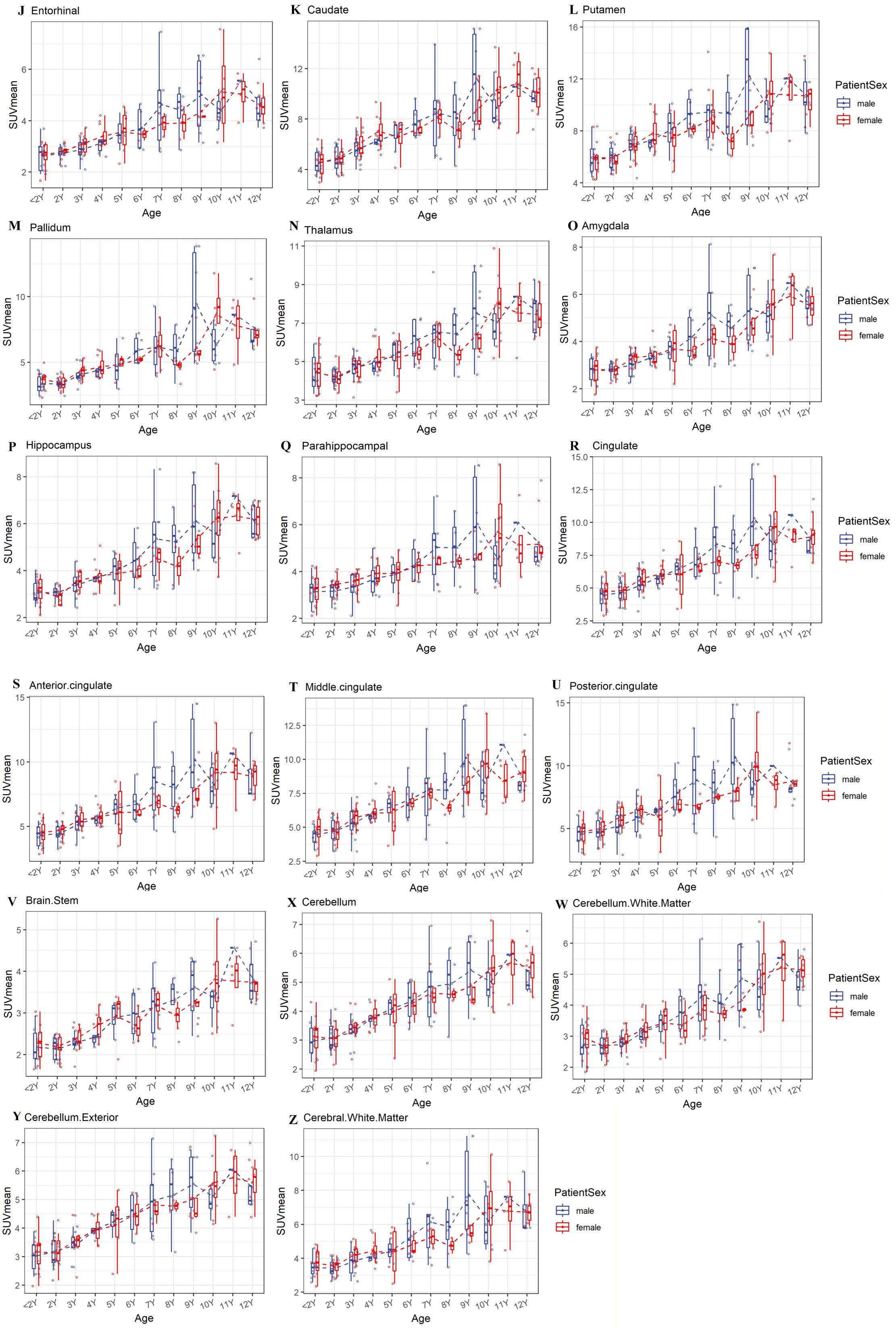
J Entorhinal
K Caudate
L Putamen
M Pallidum
N Thalamus
O Amygdala
P Hippocampus
Q Parahippocampal
R Cingulate
S Anterior.cingulate
T Middle.cingulate
U Posterior.cingulate
V Brain.Stem
X Cerebellum
W Cerebellum.White.Matter
Y Cerebellum.Exterior
Z Cerebral.White.Matter
SUVmean
Age
<2Y 2Y 3Y 4Y 5Y 6Y 7Y 8Y 9Y 10Y 11Y 12Y
PatientSex
male
female

Supplementary Figure 2: Differences in the changing trends of SUVmax values across various brain regions between genders (Two independent-samples t-test were performed for all data presented in Fig A–Z. The *T*-values ranged from −1.845 to 1.899, and *P*-values ranged from 0.082 to 0.912; all *P*-values were greater than 0.05. Each datapoint represents data from an independent single sample, and each boxplot presents the mean ± standard deviation of samples in each age group).

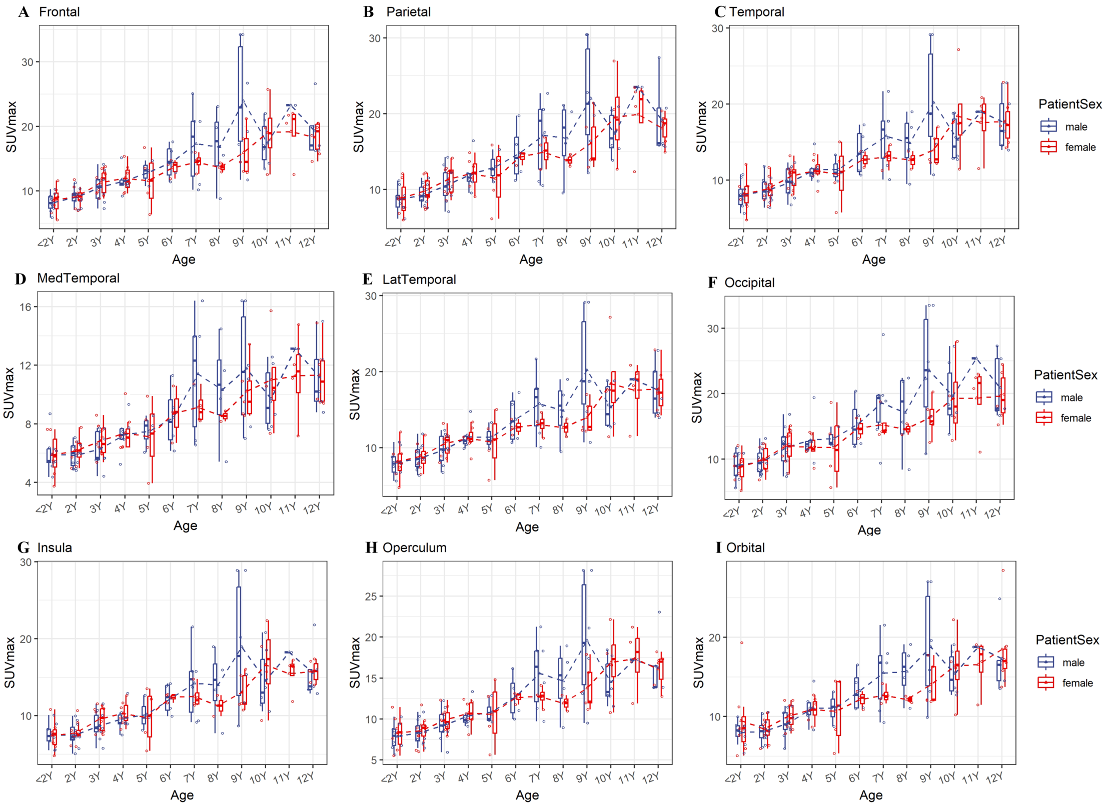

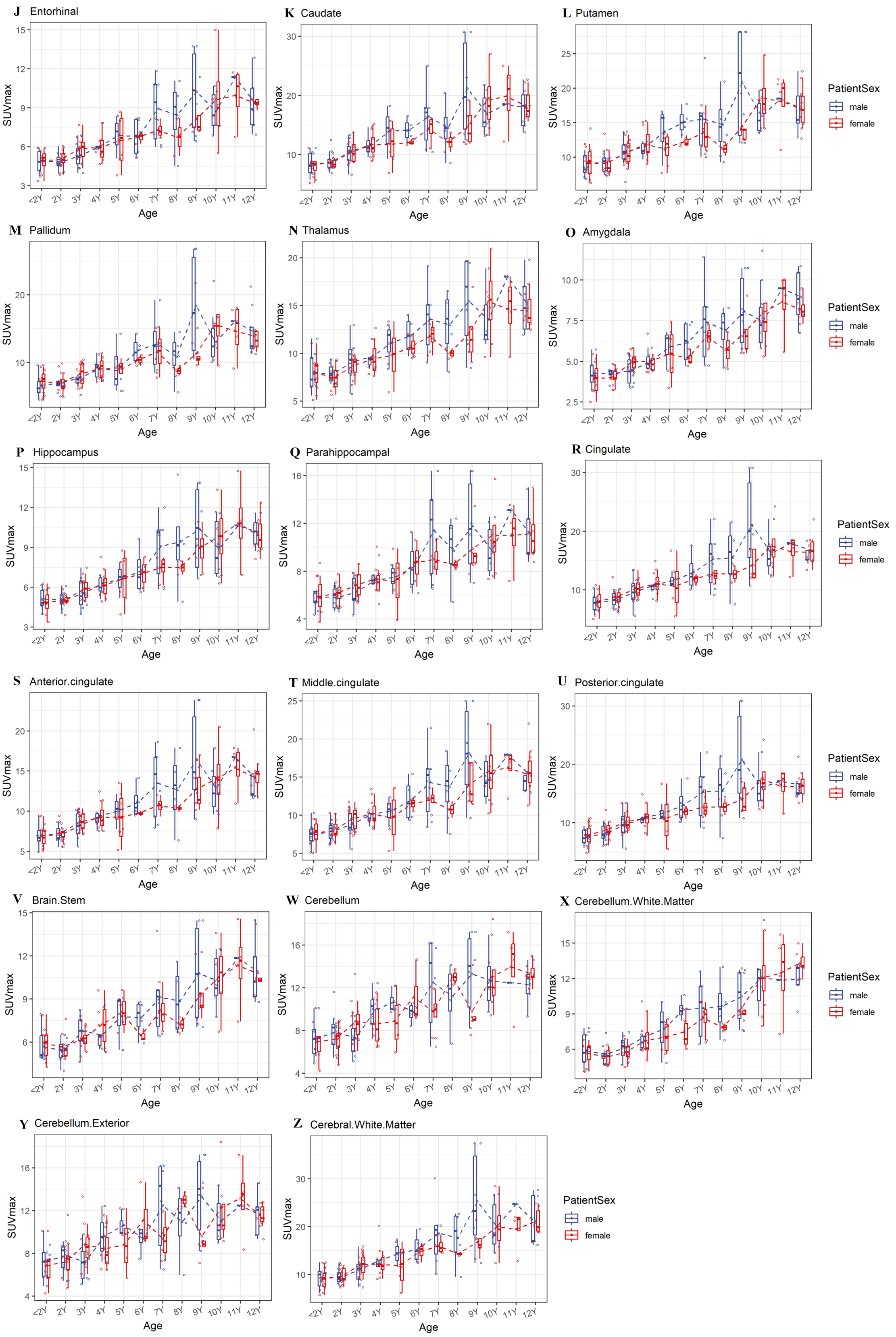

J Entorhinal
K Caudate
L Putamen
M Pallidum
N Thalamus
O Amygdala
P Hippocampus
Q Parahippocampal
R Cingulate
S Anterior.cingulate
T Middle.cingulate
U Posterior.cingulate
V Brain.Stem
W Cerebellum
X Cerebellum.White.Matter
Y Cerebellum.Exterior
Z Cerebral.White.Matter
SUVmax
Age
<2Y 2Y 3Y 4Y 5Y 6Y 7Y 8Y 9Y 10Y 11Y 12Y
PatientSex
male
female

Supplementary Figure 3: Differences in the changing trends of SUVmean values across various brain regions between left and right hemispheres (Two independent-samples t-test were performed for all data presented in Fig A–Y. The *T*-values ranged from −0.564 to 1.618, and *P*-values ranged from 0.349 to 0.994; all *P*-values were greater than 0.05. Each datapoint represents data from an independent single sample, and each boxplot presents the mean ± standard deviation of samples in each age group).

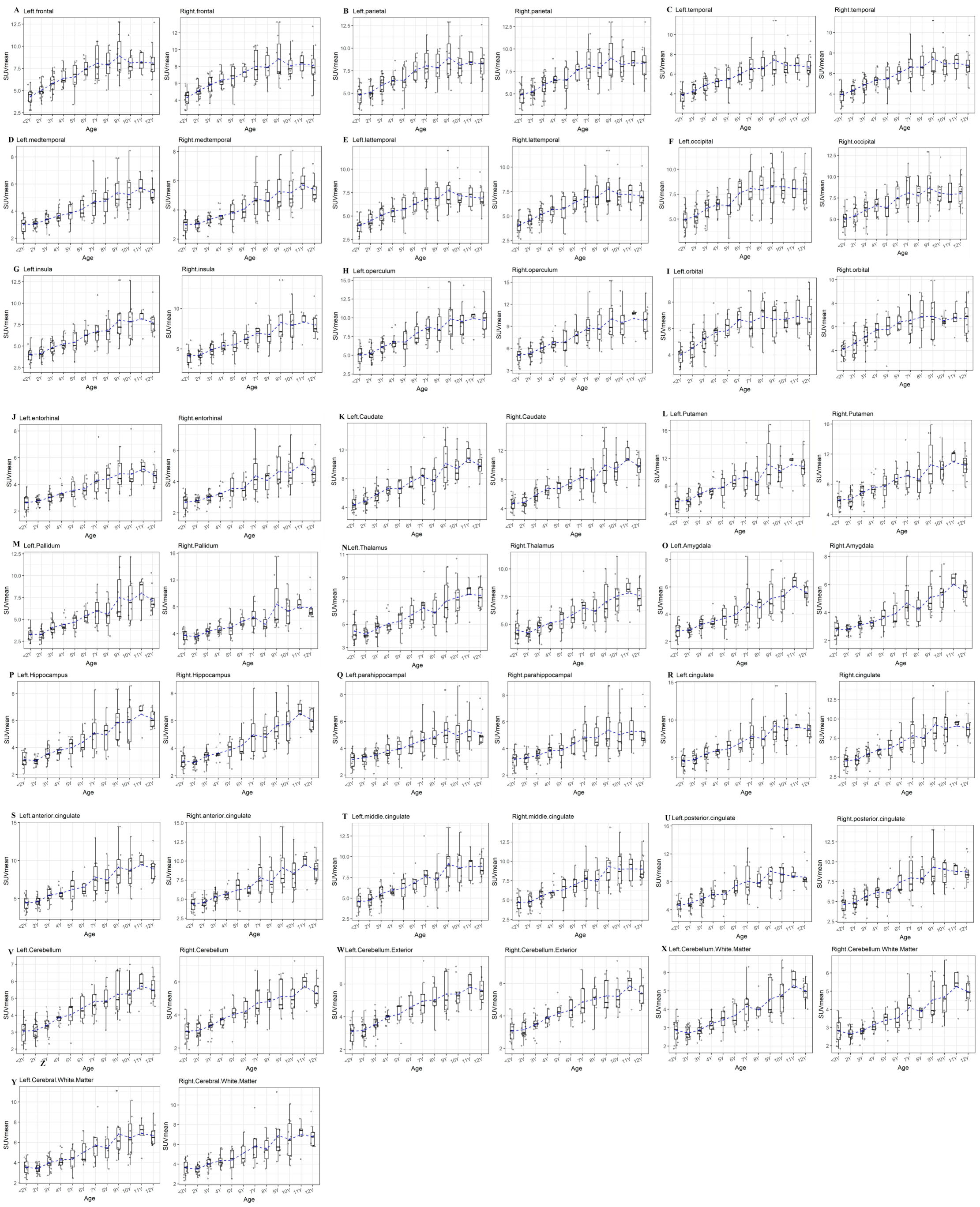

Supplementary Figure 4: Differences in the changing trends of SUVmax values across various brain regions between left and right hemispheres. (Two independent-samples t-test were performed for all data presented in Fig A–Y. The *T*-values ranged from −1.278 to 2.038, and *P*-values ranged from 0.062 to 0.997; all *P*-values were greater than 0.05. Each datapoint represents data from an independent single sample, and each boxplot presents the mean ± standard deviation of samples in each age group).

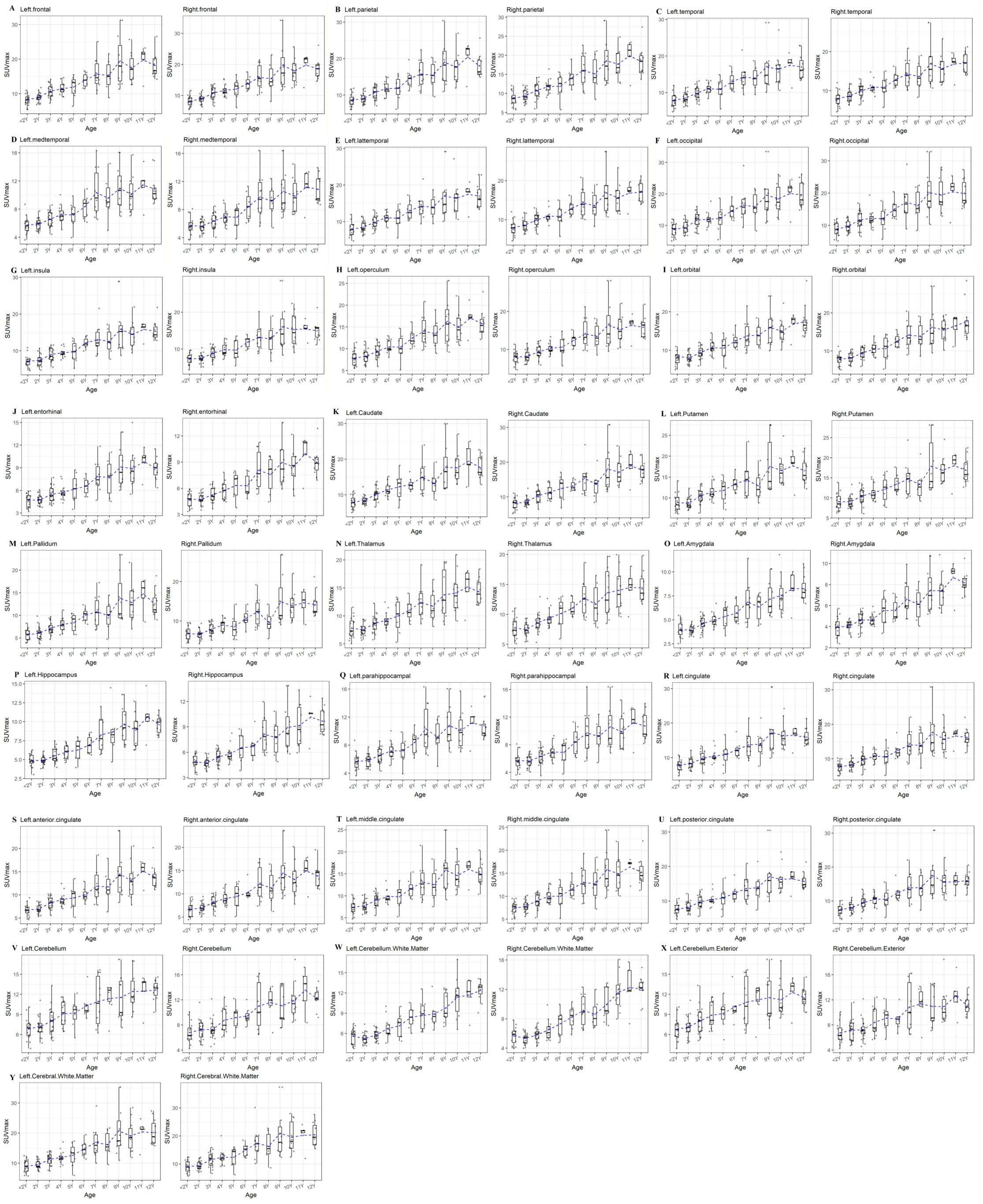

Criteria for Merging Neuromorphometrics ROI

```
Orbital = {
['Right AOrG anterior orbital gyrus']
'Right LOrG lateral orbital gyrus'
'Right MOrG medial orbital gyrus'
'Right POrG posterior orbital gyrus'
'Left AOrG anterior orbital gyrus'
'Left LOrG lateral orbital gyrus'
'Left MOrG medial orbital gyrus'
'Left POrG posterior orbital gyrus'};

Frontal = {
'Right FRP frontal pole'
'Right MFC medial frontal cortex'
'Right MFG middle frontal gyrus'
'Right MSFG superior frontal gyrus medial segment'
'Right OpIFG opercular part of the inferior frontal gyrus'
'Right OrIFG orbital part of the inferior frontal gyrus'
'Right SFG superior frontal gyrus'
'Right SMC supplementary motor cortex'
'Right TrIFG triangular part of the inferior frontal gyrus'
'Left FRP frontal pole'
'Left MFC medial frontal cortex'
'Left MFG middle frontal gyrus'
'Left MSFG superior frontal gyrus medial segment'
'Left OpIFG opercular part of the inferior frontal gyrus'
'Left OrIFG orbital part of the inferior frontal gyrus'
'Left SFG superior frontal gyrus'
'Left SMC supplementary motor cortex'
'Left TrIFG triangular part of the inferior frontal gyrus'};

Parietal = {
'Right AnG angular gyrus'
'Right MPoG postcentral gyrus medial segment'
'Right MPrG precentral gyrus medial segment'
'Right PCu precuneus'
'Right PoG postcentral gyrus'
'Right PrG precentral gyrus'
'Right SMG supramarginal gyrus'
'Right SPL superior parietal lobule'
'Left AnG angular gyrus'
'Left MPoG postcentral gyrus medial segment'
'Left MPrG precentral gyrus medial segment'
'Left PCu precuneus'
'Left PoG postcentral gyrus'
```

```
'Left PrG precentral gyrus'
'Left SMG supramarginal gyrus'
'Left SPL superior parietal lobule'};

Occipital = {
'Right Calc calcarine cortex'
'Right Cun cuneus'
'Right IOG inferior occipital gyrus'
'Right LiG lingual gyrus'
'Right MOG middle occipital gyrus'
'Right OCP occipital pole'
'Right OFuG occipital fusiform gyrus'
'Right SOG superior occipital gyrus'
'Left Calc calcarine cortex'
'Left Cun cuneus'
'Left IOG inferior occipital gyrus'
'Left LiG lingual gyrus'
'Left MOG middle occipital gyrus'
'Left OCP occipital pole'
'Left OFuG occipital fusiform gyrus'
'Left SOG superior occipital gyrus'};

LatTemporal = {
'Right FuG fusiform gyrus'
'Right ITG inferior temporal gyrus'
'Right MTG middle temporal gyrus'
'Right PP planum polare'
'Right PT planum temporale'
'Right STG superior temporal gyrus'
'Right TMP temporal pole'
'Right TTG transverse temporal gyrus'
'Left FuG fusiform gyrus'
'Left ITG inferior temporal gyrus'
'Left MTG middle temporal gyrus'
'Left PP planum polare'
'Left PT planum temporale'
'Left STG superior temporal gyrus'
'Left TMP temporal pole'
'Left TTG transverse temporal gyrus'};

roi.MedTemporal = {
'Right Amygdala'
'Right Hippocampus'
'Right Ent entorhinal area'
```

```
'Right PHG parahippocampal gyrus'
'Left Amygdala'
'Left Hippocampus'
'Left Ent entorhinal area'
'Left PHG parahippocampal gyrus'};

Amygdala = {
'Right Amygdala'
'Left Amygdala'};

Hippocampus = {
'Right Hippocampus'
'Left Hippocampus'};

Parahippocampal = {
'Right PHG parahippocampal gyrus'
'Left PHG parahippocampal gyrus'};

Entorhinal = {
'Right Ent entorhinal area'
'Left Ent entorhinal area' };

Temporal = {
'Right FuG fusiform gyrus'
'Right ITG inferior temporal gyrus'
'Right MTG middle temporal gyrus'
'Right PP planum polare'
'Right PT planum temporale'
'Right STG superior temporal gyrus'
'Right TMP temporal pole'
'Right TTG transverse temporal gyrus'
'Right Amygdala'
'Right Hippocampus'
'Right Ent entorhinal area'
'Right PHG parahippocampal gyrus'
'Left FuG fusiform gyrus'
'Left ITG inferior temporal gyrus'
'Left MTG middle temporal gyrus'
'Left PP planum polare'
'Left PT planum temporale'
'Left STG superior temporal gyrus'
'Left TMP temporal pole'
'Left TTG transverse temporal gyrus'};
'Left Amygdala'
```

```
'Left Hippocampus'
'Left Ent entorhinal area'
'Left PHG parahippocampal gyrus'};

Insula = {
'Right AIns anterior insula'
'Right PIns posterior insula'
'Left AIns anterior insula'
'Left PIns posterior insula'};

Operculum = {
'Right CO central operculum'
'Right FO frontal operculum'
'Right PO parietal operculum'
'Left CO central operculum'
'Left FO frontal operculum'
'Left PO parietal operculum'};

Cingulate = {
'Right ACgG anterior cingulate gyrus'
'Right MCgG middle cingulate gyrus'
'Right PCgG posterior cingulate gyrus'
'Left ACgG anterior cingulate gyrus'
'Left MCgG middle cingulate gyrus'
'Left PCgG posterior cingulate gyrus'};

AnteriorCingulate = {
'Right ACgG anterior cingulate gyrus'
'Left ACgG anterior cingulate gyrus'};

MiddleCingulate = {
'Right MCgG middle cingulate gyrus'
'Left MCgG middle cingulate gyrus'};

PosteriorCingulate = {
'Right PCgG posterior cingulate gyrus'
'Left PCgG posterior cingulate gyrus'};

Caudate = {'Right Caudate','Left Caudate'};
Putamen = {'Right Putamen','Left Putamen'};
Pallidum = {'Right Pallidum','Left Pallidum'};
Thalamus = {'Right Thalamus Proper','Left Thalamus Proper'};

CerebellumGray = {
```

```
'Right Cerebellum Exterior'
'Left Cerebellum Exterior'};

CerebellumWhiteMatter = {
'Right Cerebellum White Matter'
'Left Cerebellum White Matter'};

Cerebellum = {
'Right Cerebellum Exterior'
'Right Cerebellum White Matter'
'Left Cerebellum Exterior'
'Left Cerebellum White Matter'};

CerebralWhiteMatter = {
'Right Cerebral White Matter'
'Left Cerebral White Matter'};

BrainStem = {'Brain Stem'};
```
